# Chase on Making Metallic Casts, &c

**Published:** 1852-10

**Authors:** H. S. Chase

**Affiliations:** Woodstock, Vt.


*Making Metallic casts—Suction cavities in old Sets—Gilding without a battery.* By H. S. CHASE, M. D. of Woodstock, Vt.

During my dental experience I have tried a great variety of metals for casts, and have for the last two years used the following, with perfectly satisfactory results:

I mould in sand, and make one cast of zinc and one of type metal. Both should be turned into the moulds when they are on the point of solidifying. I dip the zinc cast into lead, and thus make the *counter* cast for *both* models. The plate is then "struck up" on the zinc cast perfectly, and it is so hard that it does not "batter up" in the least. But, as the zinc *shrinks in cooling,* the plate will be found too small for the mouth. Now the type metal casts comes into use; this *does not shrink* in cooling; and by "striking up" between this and the lead, as a finishing touch, you have a perfect fit, providing your "impression" was correct. Type metal *alone* will not answer, as it is too soft. I purchase of the printers their worn-out types at eight cents per pound.

*To make Suction Cavities in Old Sets.*—It is frequently desirable to make a suction cavity in a set of teeth which has none, or to enlarge the one already made. This can be done in two hours, very simply. Lay the set on a table, with the grinding surface of the teeth upwards. Oil the plate and teeth; then mix up some plaster, with salt water and a little sand, and pour it on the plate until the space bounded by the teeth is filled up a half inch above the grinding surfaces of the teeth. When the plaster is hard enough to remove without breaking, take it out, and pare away, or scoop out, that part of the plaster which comes in contact with that part of the plate where you wish to have a suction cavity. The suction cavity is to be made by pressing the plate into that part which is scooped out; therefore, cut away the plaster just the shape you wish that cavity to take, and cut into the plaster as deeply as you wish the depth of your suction cavity to be. Now replace the plaster; then mix more plaster, in the same way as before, and cover the teeth and every part of the plate with it, *excepting the part immediately above the suction cavity* which you have cut out of the first piece of plaster; lay it on to the thickness of half an inch, at least. When the whole mass is *hard*, take a small hammer, with a convex head, and strike down the plate, exposed, into the cavity, in the plaster, underneath. Do this carefully, and by gentle blows, commencing first in the centre of the plate, and advancing in a circle to the sides. Now cut away your plaster, and you have the suction cavity, and without *warping* the plate in the least. I have formed suction cavities in this way several times, and always with the most satisfactory results. It is a very valuable mode of operating in cases where there has been a shrinking of the alveolar border, and the plate rides on the roof of the mouth.

*Gilding without a Battery.*—Not long ago a professional acquaintance told me that he could gild without a battery; that he paid five dollars for the secret, and would sell it to me for that sum. I did not see fit to accept his offer, but went to experimenting, and in a short time found a way to gild without using a battery. I will now give it to the profession, for I consider it a very great convenience, and would not be without the knowledge for fifty dollars. I think there is no dentist but could use it constantly to good advantage. The occasions will suggest themselves to the minds of all.

*To make a Gilding Solution.*—Take of nitric acid one fluid drachm, of hydrochloric acid two do. do. put them together in a glass or porcelain vessel, and add sixteen (16) grains of gold, either of foil or plate. Let the gold be of the color which you wish the article gilded to partake of. If you use *plate*, cut it into small pieces. In a few hours the gold will be dissolved. Now evaporate the solution to *dryness*, and you will see a reddish substance on the bottom of the vessel, to which you may add half a pint of clean rain water. Into this put one half (½) ounce of cyanuret of potassium, which will dissolve, and then you have a *gilding solution.*

When the above quantity is prepared, a pint bowl, with perpendicular sides, is very convenient for conducting the opetion of *gilding*, which is done as follows: Clean the article to be gilded with prepared whiting, then take a clean piece of sheet zinc, the size of half a dollar, and connecting it with the article to be gilded by a small copper or platina wire, put both into the solution. In a minute or two you will see that all the metal in the solution has received a coating of gold. Now take out the article and clean it thoroughly with whiting; the *first* coat of gold will mostly come off, but every succeeding one will adhere more and more tenaciously. It is necessary, occasionally, to *remove the coating from the zinc*, otherwise the process will be arrested. If the article is left in the solution *too long* it will become dingy, and must be cleaned with whiting and placed back again. Block tin and soft solder can be gilded as well as silver or copper. If the gilding is *burnished* it will form a hard surface, which will wear a long time. The article must be polished, all that it is intended to be, *before* gilding.—*Ibid.*

